# Abnormal Expression of *COX5B* Gene and Disorder of Mitochondrial Function in Cryptorchid Rats

**DOI:** 10.1111/jcmm.70234

**Published:** 2024-11-25

**Authors:** Xuehan Wang, Dashuai Miao, Songyi Ye, Hua Xian, Wenliang Ge

**Affiliations:** ^1^ Department of Pediatric Surgery Affiliated Hospital of Nantong University Nantong China; ^2^ Department of Pediatric Surgery Medical School of Nantong University Nantong China; ^3^ Donghai County People's Hospital—Jiangnan University Smart Healthcare Joint Laboratory Donghai County People's Hospital Lianyungang Jiangsu China

**Keywords:** *COX5B* gene, cryptorchidism, mitochondrial function, TM3 cell

## Abstract

Cryptorchidism is one of the most common congenital malformations in the paediatric genitourinary tract. Data analysis of cryptorchidism‐related datasets in the GEO database and gene sequencing results from our institution, along with bioinformatic analysis of the merged mitochondrial gene datasets, revealed that *COX5B* is differentially expressed in the testes of children with cryptorchidism. Its encoded protein has attracted our attention as a key component of the mitochondrial respiratory chain complex IV. This study aims to explore the *COX5B* gene expression changes and related mitochondrial issues in cryptorchid rats. For this purpose, we established a cryptorchid rat model by surgery and used molecular biology and biochemistry techniques to detect and analyse the expression level of the *COX5B* gene and mitochondrial function indexes. The results indicated a significant decrease in *COX5B* gene expression in the affected testis of cryptorchid rats. The knockdown of *COX5B* expression in TM3 cells could be observed as the aggravation of cellular senescence, which led to the reduction of proliferation, as well as accompanied by the obvious disorders of mitochondrial function, including the increase of ROS and the decrease of ATP, in which MMP was significantly reduced. This suggests that the *COX5B* gene may play an important role in cryptorchid testis‐induced reproductive system damage and may be a new target for small molecule‐targeted therapy.

## 1 Introduction

During normal development, the testes descend from the lumbar retroperitoneum to the scrotum. The absence or incomplete descent, with no testes in the scrotum or with testes on only one side, is called cryptorchidism. Cryptorchidism is one of the most common congenital anomalies of the genitourinary tract in paediatric patients. The prevalence of cryptorchidism is 1% and still on the rise [1]. At the same time, because the testicles remain in the abdominal cavity or inguinal canal for a long time, subject to the influence of the body's ‘high temperature’, easy to cause male infertility [2]. Furthermore, it can lead to malignant changes in testicular cells due to altered growth conditions and developmental disorders, causing the formation of cancerous tumours, which is approximately 30–50 times more likely to occur [3]. The treatment options for cryptorchidism up to now mainly include surgical treatment as well as hormonal therapy. Immobilisation of the testis in the scrotum to reduce the risk of male infertility and testicular malignancy before irreversible pathological damage occurs is the main therapeutic principle, so surgical treatment is still considered to be the first choice in the clinical treatment of cryptorchidism [4, 5]. Moreover, it is believed that initiating testicular fixation at an early stage can be advantageous for the restoration of the functionality of testicular interstitial cells. Research has indicated a slight inverse correlation between the age at which testicular fixation is performed and the levels of testosterone in adulthood among males who have undergone surgery for unilateral cryptorchidism [6]. In spite of this, such surgery does not repair the original pathologic changes; a number of patients who have undergone surgical treatment in their early years develop reduced reproductive function in adulthood [7]. Therefore, it is important to explore the pathogenesis of cryptorchidism and search for possible therapeutic targets to guide the treatment of cryptorchidism as well as to minimise the occurrence of related complications.

Leydig cells, which are pivotal in the testis for the production of androgens, also play a crucial role in the maturation process of spermatogenic cells and in upholding overall reproductive health [8]. These cells are recognised for their secretion of testosterone, a hormone that is vital for metabolic regulation and the preservation of sexual function. This secretion is facilitated by the collaborative efforts of mitochondria and the smooth endoplasmic reticulum [8]. Mitochondria, often referred to as the ‘powerhouses’ of the cell, generate ATP through the process of oxidative phosphorylation and are integral to a spectrum of physiological and pathological phenomena, such as apoptosis, inflammation, oxidative stress, neuronal disorders, oncogenesis and ageing [9]. Research has shown that mitochondria play an important role in the health, proliferation and apoptosis of germ cells as well as testicular mesenchymal cells. Regarding this, many studies have focused on the link between mitochondrial function and androgen synthesis in Leydig cells [10]. It has also been shown that variations in mitochondrial DNA (mtDNA) are also associated with effects on germ cell function, and experiments have found that mtDNA haplogroups in whites are associated with azoospermia [11]. Parallel findings were observed in the examination of sperm from men with infertility and mitochondrial disorders, where numerous deletions in the sperm's mtDNA were detected [12]. It is interesting to note that a fertility assessment of British individuals with mitochondrial disorders revealed a fertility rate that was 35% lower than that of the broader population [13]. This fertility reduction was not observed in corresponding studies involving women with mitochondrial disorders. This discrepancy appears to corroborate the theory that male reproductive health is more susceptible to the effects of mitochondrial impairment linked to mtDNA irregularities.

It is worth pondering whether cryptorchidism causes mitochondrial damage in testicular mesenchymal cells and how it affects it. Research in this area is still lacking. From a biochemical point of view, cryptorchidism may lead to abnormally high testicular temperatures, causing oxidative stress in germ cells and thus affecting mitochondrial function [14]. Implications may include decreased activity of mitochondrial respiratory chain enzymes, disruptions to the mitochondrial membrane's potential, and an escalation in the production of reactive oxygen species (ROS) [15]. Such alterations may trigger the initiation of apoptosis through the mitochondrial pathway, particularly by activating the pro‐apoptotic protein Bak, an antagonist of Bcl‐2, and by interacting with the protein Bax. This interaction facilitates the permeabilisation of the outer mitochondrial membrane, leading to the release of apoptotic initiators like cytochrome c and other factors that induce cell death [16]. Oxidative phosphorylation (OXPHOS) provides bioenergy to sustain the progression of intracellular metabolic pathways to maintain cell proliferation and survival [17], five complexes comprise the OXPHOS machinery in mitochondria, complex I (NADH: ubiquinone oxidoreductase), complex II (succinate dehydrogenase), complex III (cytochrome bc1 complex), complex IV (cytochrome c oxidase, COX) and complex V (ATP synthase) and all of them are located in the inner mitochondrial membrane. Notably, among these complexes, COX (complex IV) is recognised as a key site for OXPHOS regulation [18]. The protein encoded by the *COX5B* gene is a minor structural component of the cytochrome C oxidase complex, which is located on the inner mitochondrial membrane and plays a crucial role in the last stage of the respiratory chain process. Its associated pathways include respiratory electron transport, ATP synthesis via chemical coupling and heat generation via uncoupling proteins.

Recently, understanding of mitochondria's various roles has expanded rapidly, along with advanced treatments for related diseases. In this study, we aimed to investigate the associated mitochondrial damage triggered by cryptorchidism and the mechanism of mitochondrial gene aberrant expression in cryptorchidism. To this end, we investigated the aberrant low expression of *COX5B* in a rat model of cryptorchidism, and subsequently low expression of *COX5B* in Leydig cells was observed to decrease cell proliferation and increase apoptosis, and impaired the metabolic function of mitochondria. It would provide a new perspective on our understanding of the disease and provide guidance for the prevention and treatment of related disorders.

## 2 Materials and Methods

### 2.1 Identification of Mitochondria‐Associated Cryptorchid Differentially Expressed Genes (MitoDEGs)

Information on every microarray was retrieved from GEO utilising the R package ‘GEO query’. The cryptorchid‐related datasets (GSE16191, GSE25518) were downloaded from the GEO database. DEGs from every microarray were acquired with R package ‘limma’ as executed by GEO2R online utility (https://www.ncbi.nlm.nih.gov/geo/geo2r/), and all recognised DEGs satisfied *p* < 0.05 and |log_2_ (Fold‐change)| ≥ 1. Resultant DEGs were portrayed by Volcano Plot utilising R package ‘ggplot2’ [19]. As well as the sequencing results of clinical cryptorchid specimens from the Affiliated Hospital of Nantong University. Sequenced samples were collected from nine paediatric patients who had surgical procedures in the hospital's Paediatric Surgery Department between January 2016 and June 2018. This group comprised six patients diagnosed with cryptorchidism, two with testicular injuries, and one with an incarcerated hernia who also underwent an open testicular biopsy. The collection of samples was conducted with the informed consent of the patients and their legal guardians, and all ethical approvals were in place. The children were in optimal health and had no prior surgical history. The samples were obtained with informed consent and ethical approval from the affected parties and their guardians, and the children were in good health with no history of surgery. The samples were analysed by differential analysis using the R language and merged into cryptorchid differentially expressed genes (DEGs). Mitochondrial genes were obtained from the MitoCarta 3.0 database (http://www.broadinstitute.org/mitocarta) [20], and analysed by Venn diagram with DEGs to obtain mitochondria‐associated cryptorchid differentially expressed genes (MitoDEGs), and the Venn diagrams were plotted by using an online tool.

### 2.2 Functional Enrichment Analysis of DEGs

To analyse the functional enrichment of gene sets, we first downloaded the c5.go.bp.v7.4.symbols. Utilising the Molecular Signatures Database as a reference (DOI: 10.1093/bioinformatics/btr260, http://www.gsea‐msigdb.org/gsea/downloads.jsp), genes were aligned with the database's gene set. Subsequently, an enrichment analysis was conducted employing the R package clusterProfiler (version 3.14.3), to derive the gene set enrichment outcomes. The parameters for gene set size were established with a minimum of 5 and a maximum of 5000. Statistical significance was determined by a *p* value threshold of < 0.05 and a FDR of < 0.1.

### 2.3 Protein–Protein Interactions (PPI) Network Analysis and Identification of Hub Genes

PPI analyses for overlapping MitoDEGs were performed using the STRING database (https://string‐db.org/), and the resulting interactions were presented as a network, as implemented by Cytoscape 3.8.2. The hub MitoDEGs were selected using the CytoHubba and MCODE plugins in Cytoscape 3.8.2.

### 2.4 Animals

Male 28‐day‐old SD rats were acquired from the Experimental Animal Center of Nantong University. Maintain suitable indoor ring conditions with the temperature at 18°C–22°C and a 12 h day‐night cycle. All procedures involving animals were in compliance with the Basel Declaration and with the Regulations of the People's Republic of China on the Administration of Laboratory Animals, and ethical approval was granted by the Animal Care and Use Committee of Nantong University (IACUC20220210‐1001).

### 2.5 Surgical Cryptorchidism Mouse Model

Five‐week‐old male SD rats were administered anaesthesia via an intraperitoneal injection of 3% sodium pentobarbital at a concentration of 0.15 mL/Kg of body weight. Surgical intervention was carried out in the right inguinal area of the experimental rats, where the right testis was extracted into the abdominal cavity and affixed to the abdominal wall with sutures. In contrast, the left testis remained in its natural position within the scrotum. For the control group, a similar incision was made but without the manipulation of the right testis, simulating the surgical procedure. Postoperatively, after a 25‐day recovery period, the rats were euthanised, and their testes were harvested for subsequent experimental analysis.

### 2.6 Testicular Tissue Acquisition and HE Staining

The rats were anaesthetised with an intraperitoneal injection of 1.5 mL/kg sodium pentobarbital, then the abdomen was cut open by ophthalmic scissors, with emphasis on exposing the bilateral testes and bladder, and the testes were taken from both sides of the mouse, the testicular leukomalacia was peeled off, and the testes were placed into 4% paraformaldehyde solution for specimen fixation, and after sufficient fixation, the specimens were dehydrated by gradient of different concentrations of ethanol solution, and paraffin embedding was carried out after dehydration, and the embedded specimens were sliced along the transverse axes of the testes. The morphological changes of testicular tissue were observed microscopically using haematoxylin–eosin staining, and the results were analysed by videotaping using a Nikon optical microscope.

### 2.7 Immunohistochemical (IHC) Staining Analysis of Testicular Tissue

Testicular tissue sections were grilled and baked separately, deparaffinised in xylene, and after gradient alcohol immersion, then antigenically repaired using sodium citrate, followed by overnight incubation with *COX5B* (1:300, TN23972S, Abmart, Shanghai, China) antibody at 4°C. Three times rinsed by TBST, dropwise addition of secondary antibody (enzyme‐labelled anti‐mouse/rabbit IgG polymer), incubation at 25°C for 30 min, wash out three times by TBST, stained by DAB, re‐stained by haematoxylin, dehydrated and finally sealed.

### 2.8 Protein Extraction

The mouse testis tissues were put into pre‐cooled EP tubes, appropriate amount of RIPA lysis solution was added, and the tissues were ground to homogenisation by using a high‐throughput tissue grinder, centrifuged for 30 min at 4°C, 12,000 rpm, the supernatant was aspirated into new pre‐cooled EP tubes, and the total protein concentration was determined according to the steps in the instruction manual of the BCA Protein Concentration Determination Kit (Biosharp, Hefei, China), and the protein was denatured and stored at −20°C.

### 2.9 Western Blot Analysis

After taking 30 μg of each sample for electrophoresis according to SDS‐PAGE gel (Epizyme PG114), it was transferred to a PVDF membrane, blocked by protein‐free rapid blocking buffer (Epizyme PS108P), added with rabbit anti‐*COX5B* (1:3000, TN23972S, Abmart, Shanghai, China) and mouse anti‐β‐Actin (1:5000, Epizyme, Shanghai, China), respectively, and placed on a shaking bed at 4°C overnight. Incubate the rabbit and mouse secondary antibodies (1:10,000, Epizyme, Shanghai, China) at room temperature for 1 h respectively, and then expose the developed strips after the luminescent solutions A and B were dispensed at a ratio of 1:1 to avoid light according to the instructions of the ECL luminescent kit; analyse the images and make graphs and statistics by using Image J and GraphPad Prism 8.0.1 software.

### 2.10 Cell Culture

The mouse Leydig cell line TM3 was from Cell Bank, Chinese Academy of Sciences (Shanghai, China) (Shanghai Institute of Bioscience, China). TM3 cells were routinely cultured in DMEM/F‐12 culture medium containing 5% horse serum (Zhongqiaoxinzhou Biotech, Shanghai, China) and 2.5% fetal bovine serum (Zhongqiaoxinzhou Biotech, Shanghai, China) at 37°C in a 5% CO_2_ incubator.

### 2.11 ShRNA Against *COX5B* and Transfection

The expression of *COX5B* was knocked down in TM3 cells by using the *COX5B*‐targeting shRNA. The shRNA molecules were synthesised and annealed by Tsingke Biotechnology in Jiangsu, China, and were then purified and inserted into the pLV‐shRNA vector. Recombinant lentiviral particles were created by co‐transfecting this vector with pMD2.G and psPAX2 plasmids into HEK293T cells. The target sequences of the shRNA for *COX5B* were as follows: (1). 5′‐GAAGTCGATGCTGGTGCTTCT‐3′; (2). 5′‐GCTTTAAACACCCTTCACATA‐3′; and (3). 5′‐CGCTCAGAAACCAAATTAAAC‐3′. TM3 cells were incubated with lentivirus and polybrene (5 μg/mL) for 24 h, followed by the addition of puromycin (5 μg/mL) to the medium to select the transduced cells.

### 2.12 ROS and ATP Quantification Assays

To quantify cellular reactive oxygen species (ROS) and ATP levels, assay kits (Elabscience, Beyotime, Wuhan, Shanghai, China, E‐BC‐K138‐F for ROS and S0026 for ATP) were used and assayed according to protocols provided by the manufacturer.

### 2.13 Apoptosis Detection Assay

TM3 cells were obtained by previously established methods. Apoptosis of the cells was measured using the Annexin V‐APC/7‐AAD Apoptosis Kit (E‐CK‐A218, Elabscience, Wuhan, China) as per the manufacturer's guidelines. Flow cytometry (BD LSRFortessa, USA) was utilised to assess cell apoptosis.

### 2.14 Mitochondrial Membrane Potential Assay

To detect Mitochondrial Membrane Potential (MMP), cells were stained with 2 μM JC‐1 for 20 min at 37°C in a dark environment using the Mitochondrial Membrane Potential Assay Kit JC‐1 (Beyotime, Shanghai, China). Then the cells were photographed with a fluorescence microscope.

### 2.15 Cell Counting Kit 8 (CCK‐8) Assay

Cell viability was determined using a cell proliferation kit (Biosharp, Hefei, China). Cells were inoculated into 96‐well plates at a density of 2 × 10^3^ cells/well and incubated with CCK‐8 reagent every 24 h for 2 h at 37°C for a total of 4 days. Cell viability signals were detected at 450 nm using an enzyme‐linked immunoassay detector.

### 2.16 Statistical Analysis

SPSS 26.0 was used for statistical analysis, Image J for Western Blot grey value determination, and GraphPad Prism for graphing. *t*‐tests were used for group comparisons, considering *p* < 0.05 statistically significant.

## 3 Results

### 3.1 Identification of MitoDEGs Associated With Cryptorchidism

**FIGURE 1:**
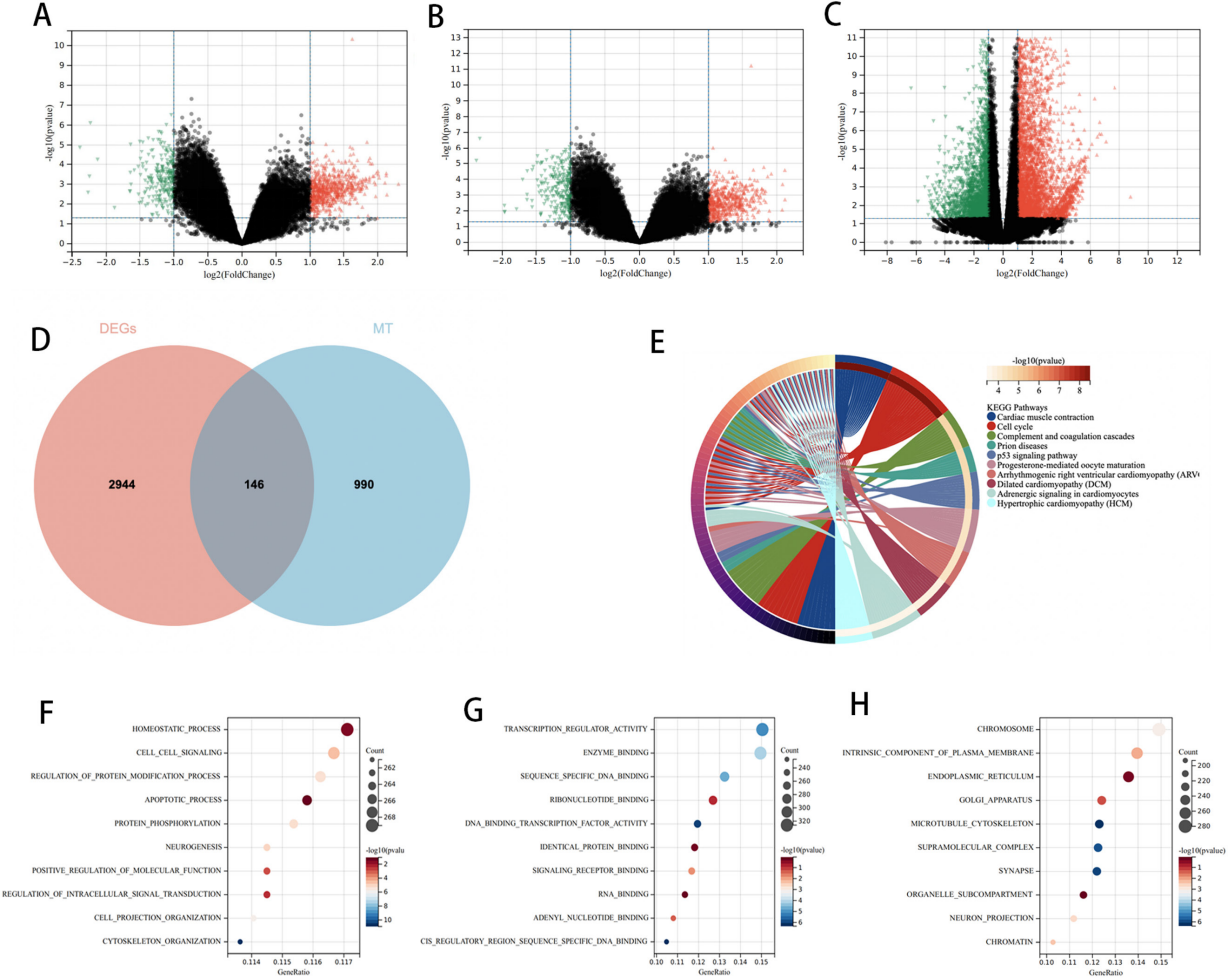
Screening and identification of MitoDEGs. (A–C) The differentially expressed genes of GSE16191, GSE25518, RNA‐Seq. The horizontal axis is the fold change in differential gene expression, the vertical axis is the *p*‐value, and the two vertical dashed lines are the differential gene expression fold thresholds, DEGs were selected with a fold change > 2 and *p* value < 0.01, with up‐regulated genes indicated by red dots and down‐regulated genes indicated by green dots. (D) Wayne diagram showing the overlap of DEGs and MitoDEGs. (E) KEGG pathway enrichment results in MitoDEGs. (F–H) The enriched GO terms of MitoDEGs.

Through a comparative analysis of gene expression in UDT and DT tissues from the cryptorchidism‐related gene dataset, a total of 682 DEGs were identified in the GSE16191 dataset post‐normalisation of microarray data. This included 483 genes that were up‐regulated and 199 that were down‐regulated. In the GSE25518 dataset, 601 DEGs were identified, comprising 403 up‐regulated genes and 198 down‐regulated genes. Concurrently, differential expression analysis was performed on gene profiles from cryptorchid samples derived through sequencing, yielding 2517 DEGs after normalisation. Among these, 1144 genes exhibited up‐regulation, while 1373 genes showed down‐regulation. As visualised in the form of volcano plots (Figure 1A–C), the differentially expressed genes obtained from the three datasets were combined to obtain DEGs. Meanwhile, mitochondria‐associated genes were searched from the MitoCarta3.0 database, and genes overlapping with the DEGs among the mitochondria‐associated genes were selected to serve as mitochondria‐associated cryptorchid testis differentially expressed genes (MitoDEGs), which yielded 146 overlapping MitoDEGs (Figure 1D).

The Molecular Signatures Database was employed to conduct a functional and pathway enrichment analysis on the DEGs to elucidate their biological roles. The most significantly enriched KEGG pathways associated with the MitoDEGs were primarily those linked to cell cycle regulation, P53 signalling, apoptotic processes, phagosome function and cellular senescence, among others (Figure 1E). The most enriched GO terms were classified as Biological Process (BP), Cellular Component (CC) and Molecular Function (MF). It showed that MitoDEGs were mainly involved in the process of self‐equilibration of biological processes, cell signalling, regulation of protein modification processes and apoptotic processes (Figure 1F). MitoDEGs predominantly exhibited enrichment in molecular function categories such as acting as transcriptional regulators, binding to enzymes, engaging in sequence‐specific DNA binding, interacting with nucleotides, modulating the activity of DNA‐binding transcription factors, binding to identical proteins, associating with signalling receptors and binding to adenosine nucleotides (Figure 1G). Primarily enriched in chromosomes, intrinsic components of the plasma membrane, endoplasmic reticulum, Golgi apparatus, microtubule cytoskeleton and synapses (Figure 1H).

### 3.2 Hub MitoDEGs Identification and PPI Network Analysis

**FIGURE 2:**
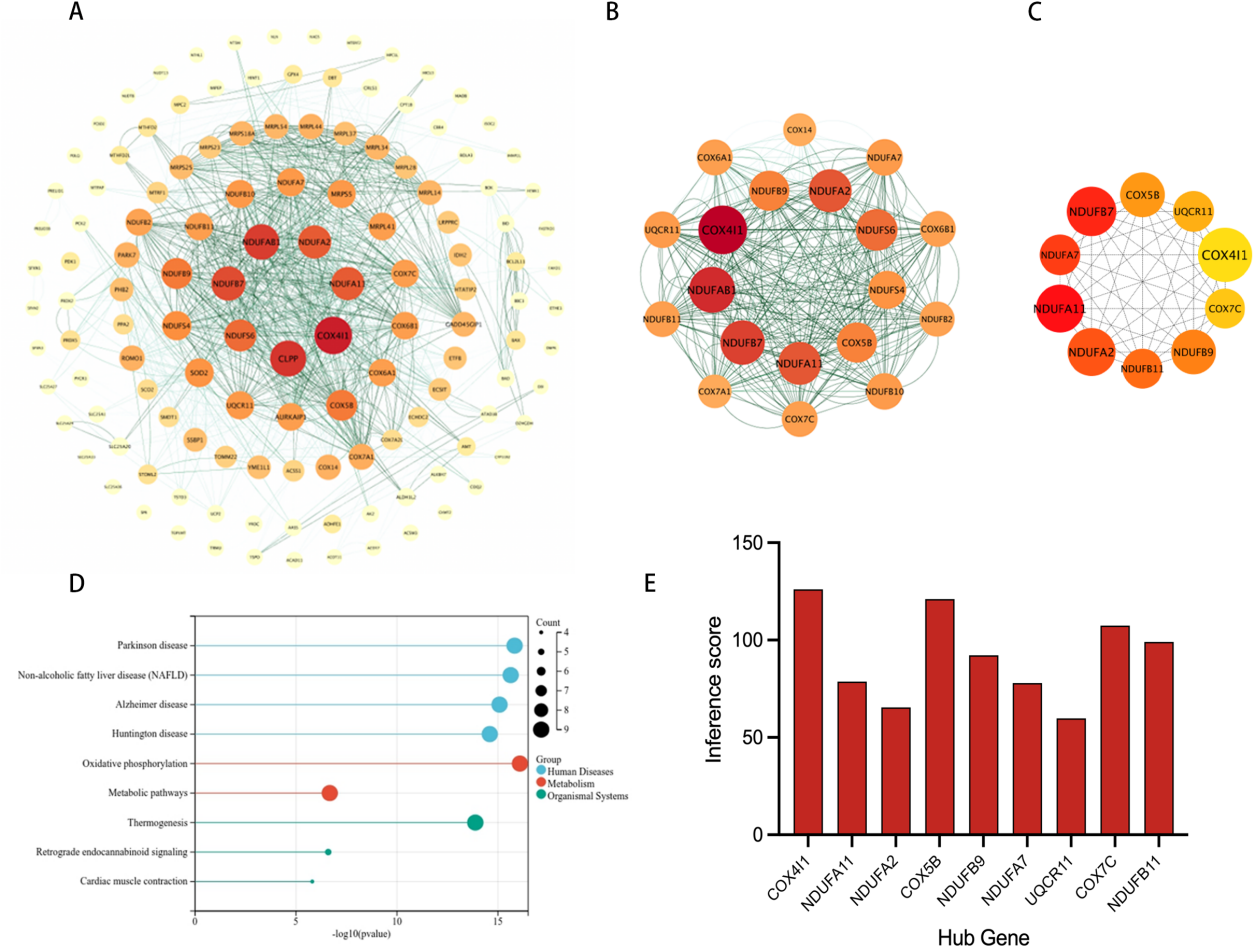
PPI network analysis; Relationship between hub MitoDEGs and cryptorchidism. (A) PPI network of MitoDEGs; (B) A key cluster with 19 genes was further chosen by MCODE; (C) Top 10 hub genes explored by CytoHubba; (D) KEGG pathway enrichment results in hub MitoDEGs; (E) Hub MitoDEGs related to DCM and HF diseases based on the CTD database.

The PPIs of 146 MitoDEGs were examined utilising the STRING database, and the data were graphically represented as a network in Cytoscape (Figure 2A). Key modules (clusters of genes) within the network were extracted using the MCODE plugin, which is integrated into Cytoscape, with specific parameters set as follows: degree cutoff = 2; node score cutoff = 0.2; k‐core = 2; and maximum depth = 100. A significant module, comprising 19 nodes and 334 edges, was detected, as well as genes involved in the module were COX4I1, NDUFAB1, NDUFB, NDUFA11, NDUFA2, NDUFS6, *COX5B*, NDUFB9, NDUFS4, NDUFA7, NDUFB10, UQCR11, COX6B1, COX7C, NDUFB11, COX6A1, NDUFB2, COX7A1, COX14 (Figure 2B). Ten candidate hub genes were identified from the PPI network using the MCC algorithm in the CytoHubba plugin, including NDUFA2, NDUFA11, NDUFB9, *COX5B*, UQCR11, COX7C, COX4I1, NDUFA7, NDUFB11, NDUFB7 (Figure 2C). Combining the results, nine hub MitoDEGs were finally obtained, including COX4I1, NDUFA11, NDUFA2, *COX5B*, NDUFB9, NDUFA7, UQCR11, COX7C, NDUFB11. Meanwhile, KEGG enrichment analysis of the obtained key MitoDEGs showed that they mainly acted on oxidative phosphorylation, metabolic pathways, thermogenesis, retrograde endogen‐ous cannabinoid signalling and myocardial contraction (Figure 2D). It was interesting to note that the resulting hub genes were all lowly expressed in the bioinformatic analyses done in the previous period, which once again shows that in cryptorchidism, the mitochondrial function of the testis is impaired. T The expressions and signalling pathways of key genes in various tissues were queried on The Human Protein Atlas website (https://www.proteinatlas.org), selecting those highly expressed in the testis. Additionally, the CTD database was used to predict the relationship between hub MitoDEGs and testicular diseases (Figure 2E). The analysis shows that *COX5B* is highly expressed in the testis and has the highest correlation with testicular disease.

### 3.3 Surgery‐Induced Cryptorchidism Leads to a Reduced Testicular Size and Weight

**FIGURE 3:**
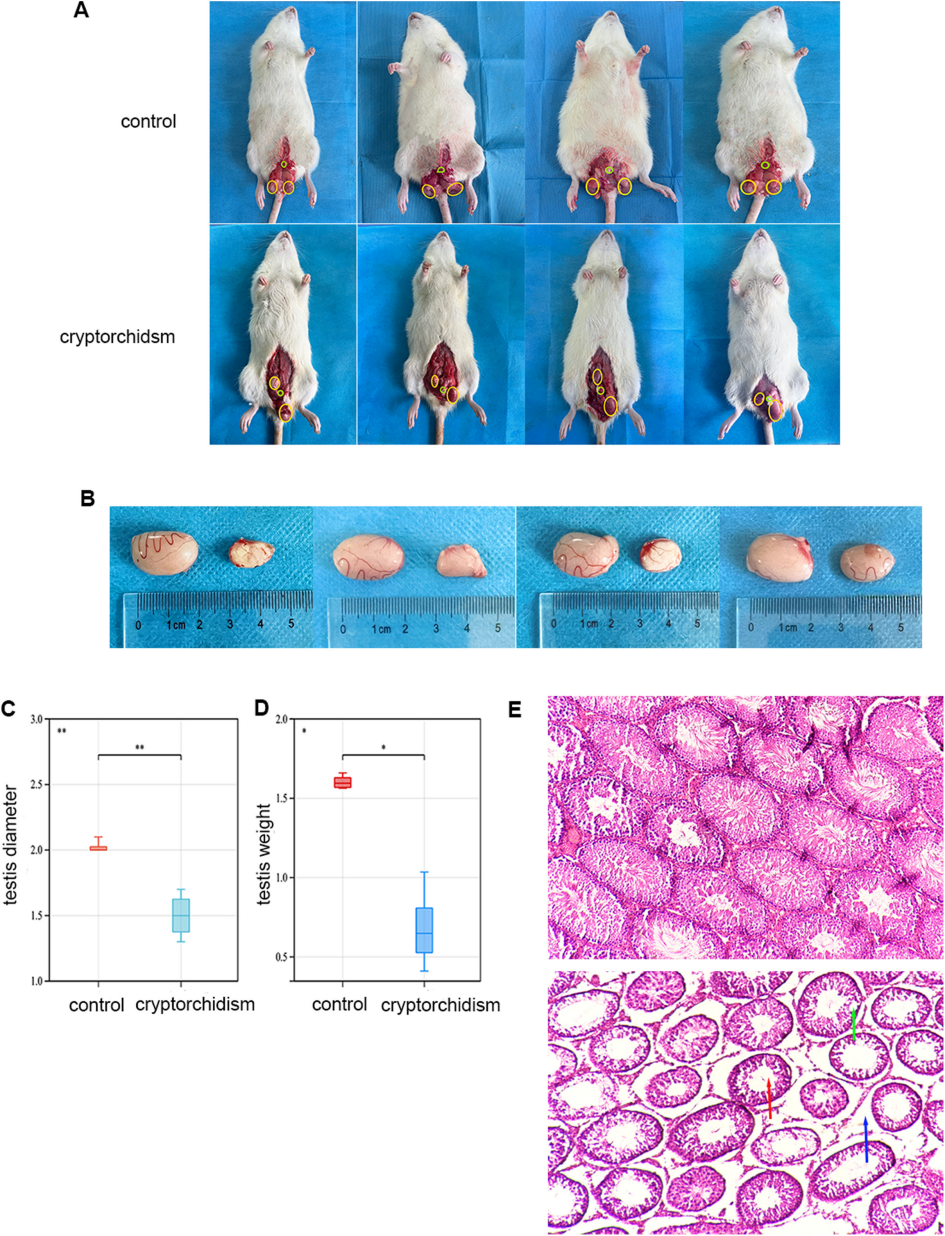
Testicular histomorphologic and structural changes in rats modelled for cryptorchid surgery. (A) Testicular position of control and cryptorchid rats after modelling; (B, C) show a comparison of testicular size. (D) Comparison of testicular weights; (E) HE staining of testicular sections. Red arrows indicate enlarged lumen of the seminiferous tubules, and green arrows indicate thickening of the border membrane of the seminiferous tubules with marked vesicular degeneration. Blue arrows show enlarged intercellular spaces. *n* = 4, *p < 0.05, **p < 0.01.

First, we constructed a right‐sided cryptorchid rat model (Figure 3A), and after 4 weeks of surgical modelling, the testes of cryptorchidism and control rats were removed respectively, and a significant decrease in testicular weight and size was found in cryptorchidism group rats (Figure 3B–D). HE staining of the samples was performed to observe the histological changes, and the results show that the testes of the cryptorchidism group lost most of the spermatogenic epithelial cells except spermatogonia and supporting cells. The diameter of the seminiferous tubules was significantly reduced, the tube lumen was enlarged, the fibrosis in the peritubular and perivascular areas was significantly increased, and the intercellular interstitial space was increased (Figure 3E). These findings align with the prior results reported by Kerr, Rich and Kretser [21] in a rat model of cryptorchidism.

### 3.4 *COX5B* Level Is Down‐Regulated in the Testis of Cryptorchid Rats

**FIGURE 4:**
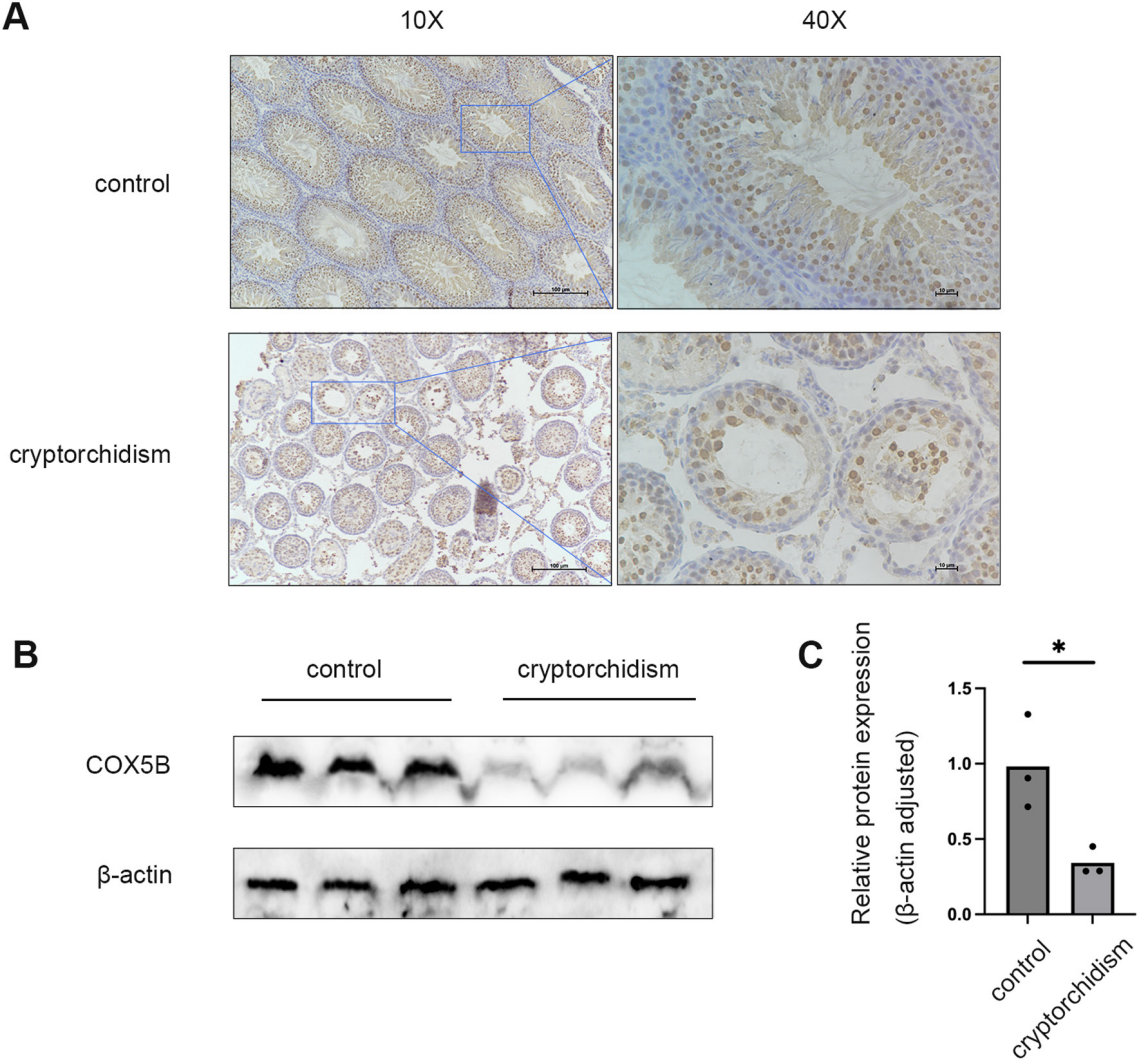
The expression level of *COX5B* in the testis. (A) Immunohistochemical staining of *COX5B* in the testes of control and cryptorchidism mice; (B) Western blot analysis of *COX5B* expression in the testicular tissues; (C) Measurement of grey‐scale statistics of *COX5B* expression in testicular tissues of mice in each group. *n* = 3, *p < 0.05.

In order to substantiate the bioinformatic analysis and to assess the relationship between COX5B protein levels and patient outcomes, we measured COX5B expression in the right testis of rats from both the cryptorchid and control groups using IHC and Western blotting techniques. The study outcomes aligned with our hypotheses, revealing a notably reduced expression of COX5B in the testes of the affected side in the cryptorchid group (Figure 4).

### 3.5 The Low Expression of *COX5B* Inhibits Proliferation by Promoting Apoptosis in Leydig Cells

**FIGURE 5:**
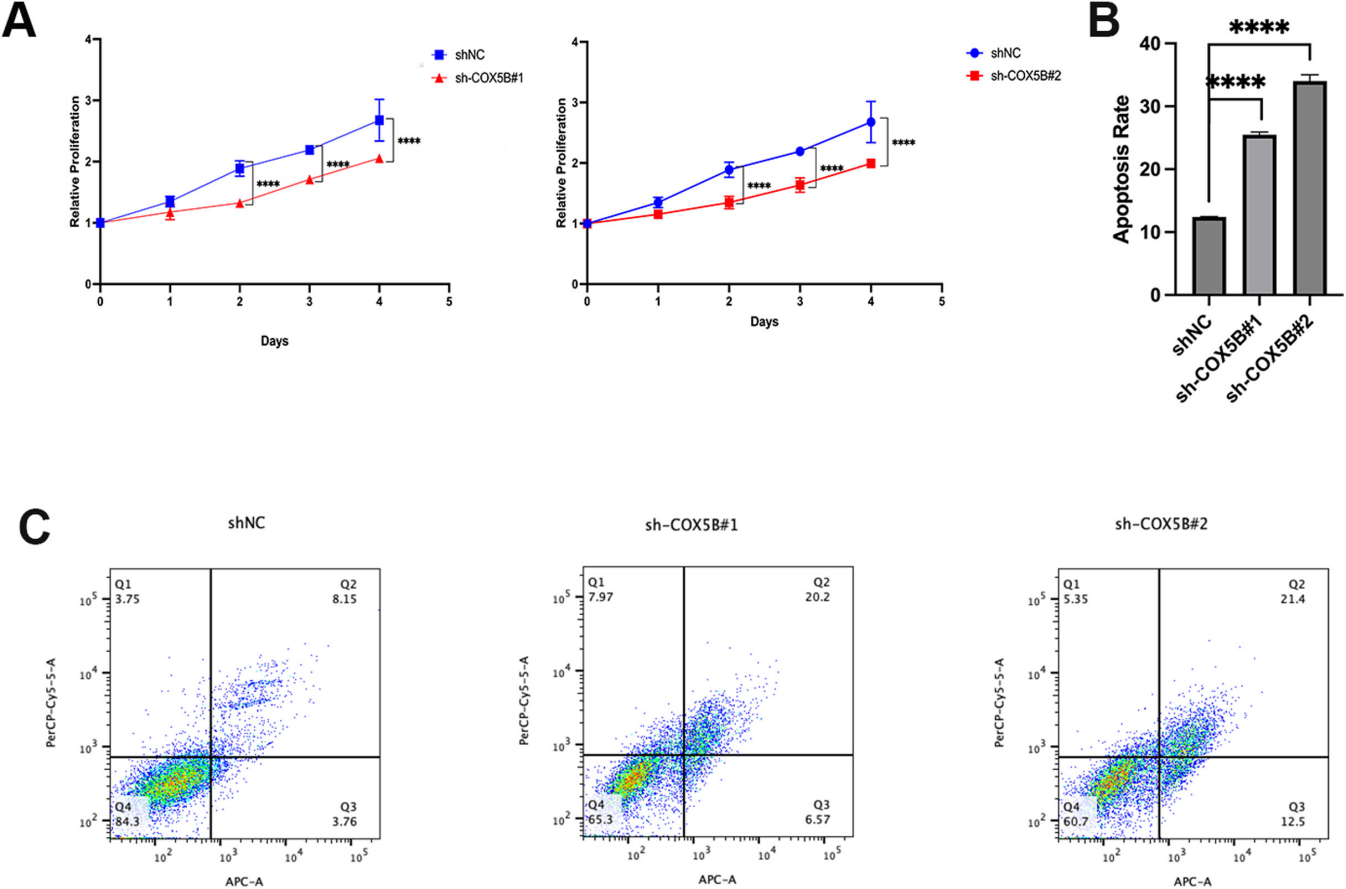
Deletion of COX5B inhibited Leydig cell proliferation but induced apoptosis. (A) The proliferation of knocked down Leydig cell lines; (B) Apoptosis rates of two stable knockout Leydig cell lines; (C) Flow cytometric analysis of apoptosis in knockdown cells. *n* = 3, ****p < 0.0001.

To investigate the role of *COX5B* in Leydig cells, the expression level of *COX5B* in Leydig cells was changed in this study. In this research, the Leydig cell line (TM3) was used to detect the impact of decreasing its level in Leydig cells on the function. As shown in Figure 5A, it was observed by the cck8 assay that an absence of *COX5B* in TM3 cells significantly inhibited cell proliferation. To explore the causes of this phenomenon, we performed cellular apoptosis assays. Knockdown of *COX5B* was performed on the TM3 cell line, stabilised, and cultured in spread plates, and the cells were collected after 72 h to observe apoptosis. The results showed that low expression of *COX5B* resulted in an elevated apoptosis rate (Figure 5B). The experimental results showed that apoptotic cells were distributed in both early and late apoptotic phases. This suggests that down‐regulation of *COX5B* affects the proliferative function of Leydig cells by promoting the apoptotic process.

### 3.6 Knock‐Down of *COX5B* Leads to Mitochondrial Dysfunction in Leydig Cells

**FIGURE 6:**
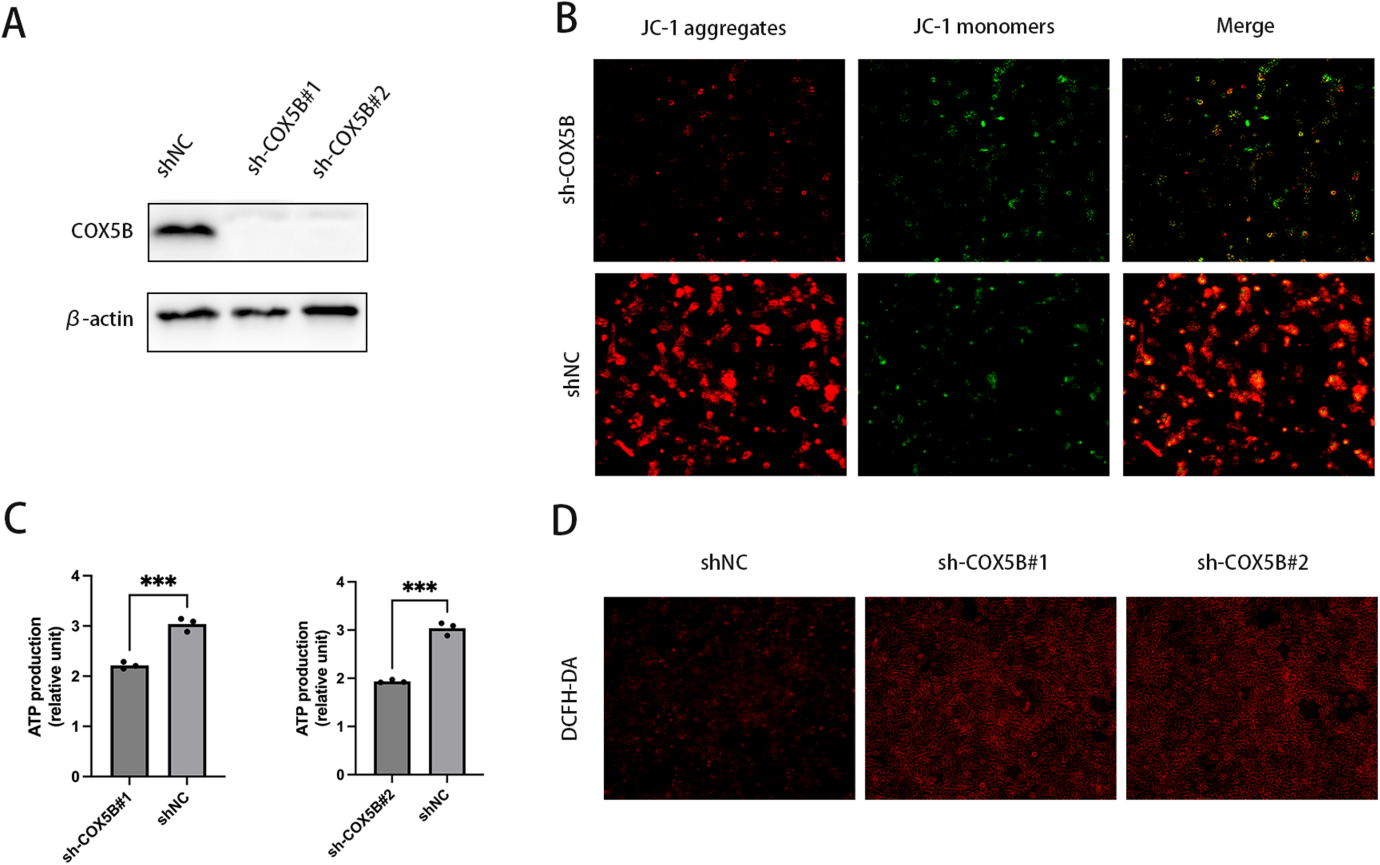
Under‐expression of *COX5B* leads to metabolic disorders in mitochondria. (A) Western blot detection of *COX5B* protein expression in stable knockout cells; (B) Quantification of mitochondrial membrane potential by JC‐1 staining; (C) Detection of ATP levels in Leydig cells with stable knockdown of *COX5B*; (D) Quantitative detection of ROS by utilising DCFH‐DA. Data were expressed as mean ± SD from three independent experiments. ***p < 0.001.

Given the significance of the *COX5B* gene‐encoded protein in the mitochondrial electron transport chain, serving as a vital component of complex IV of the mitochondrial respiratory chain, we conducted further assessments to determine the impact of *COX5B* on mitochondrial metabolism within Leydig cells. Intracellular ATP measurements revealed that the suppression of *COX5B* expression markedly decreased ATP levels in comparison to the control group (Figure 6B). Moreover, the downregulation of *COX5B* was associated with a substantial decrease in mitochondrial membrane potential and a concomitant rise in ROS levels (Figure 6C,D). Collectively, these findings suggest that the reduction of *COX5B* expression contributes to mitochondrial dysfunction within cells, potentially linking to elevated apoptotic activity.

## 4 Discussion

Cryptorchidism is a congenital abnormalities of the genitourinary system and is a common male condition that usually results in damage to the germ cells and interstitial cells of the testes. Before birth, some cryptorchid infants already have a reduced number of testicular germ cells; 23% of foetuses with cryptorchidism have fewer germ cells compared to foetuses with descending testes of the same gestational age [22]. This suggests that cryptorchidism may be an important factor in the loss of germ cells in nonviable foetuses, although this conclusion remains to be verified in live births. Hadziselimovic et al. suggest that neonatal germ cells are transformed into adult dark spermatogonia (AD‐S) early in the postnatal period, which play a key role in subsequent fertility [23]. It has been shown that cryptorchidism inhibits the conversion of gonadal cells to AD‐S, with only about half of the neonatal germ cells converting to AD‐S [24]. Meanwhile, in cryptorchid rats, Leydig cell function is impaired from 7 to 14 weeks, and its secretion of insulin‐like factor 3 (Insl3) is strongly associated with testicular descent, which may further contribute to cryptorchid formation [25]. In children, on the other hand, since testicular spermatogenesis and steroidogenesis are not fully established until puberty, this period is critical for the development of the male reproductive system. The treatment of cryptorchidism has been updated and supplemented since the discovery of the disease, and is now mainly surgical, supplemented by androgen therapy. Prior research has shown that gene expression in the testicular tissue of children with cryptorchidism differs from those with descended testes [26]. Therefore, small molecule‐targeted therapies may be effective in some patients to reduce testicular damage caused by cryptorchidism, however, additional biomarkers and targets are still needed to manage this disease. To address this problem, we established a rat cryptorchid testis model, in which the right testis of the rat was surgically fixed in the abdominal cavity at 5 weeks of age, a period when the testes of the rat begin to descend, and under normal circumstances, male SD rats become sexually mature at 60 days [27]. In order to conduct further research, the testes were removed at 60 days of age. In this study, we revealed a significant decrease in *COX5B* gene expression in the testicular tissues of cryptorchid rats, aligning with GEO database and sequencing findings. In the meantime, the knockdown of *COX5B* in the TM3 cell line promotes apoptosis and thus leads to a decrease in proliferation. It is worthwhile to explore a series of changes caused by the down‐regulation of the *COX5B* gene, which encodes a protein that is a key component of the mitochondrial respiratory chain complex IV. Earlier studies have demonstrated that elevated levels of *COX5B* in human breast and prostate cancer tissues enhance the proliferative and invasive capabilities of cancer cells. Conversely, the suppression of *COX5B* expression has been found to impede cancer cell growth by dampening mitochondrial activity, thereby curbing tumour progression [28]. Consistent with this, our results show that low *COX5B* expression in Leydig cells leads to an increase in ROS production, which reduces mitochondrial membrane potential depolarisation as well as intracellular ATP production, which subsequently leads to mitochondrial dysfunction. Specifically, flow cytometry results indicated that approximately 50% of the apoptotic cells had progressed to the late stages of apoptosis, a phenomenon predominantly attributed to oxidative stress and inflammatory responses. These findings are in line with prior research on cryptorchidism.

The decreased expression of the *COX5B* gene leads to a decrease in the activity of mitochondrial respiratory chain complex IV, which not only directly affects mitochondrial respiratory function and energy production efficiency, but also may disrupt the intracellular energy homeostasis at a deeper level [29]. Normal cellular physiological activities are highly dependent on a stable and sufficient supply of energy; insufficient energy may lead to a series of metabolic disorders, affecting protein synthesis, lipid metabolism, and other important life processes. Excessive production and accumulation of ROS lead to oxidative damage of intracellular biomolecules, including proteins, lipids and nucleic acids. This damage may further weaken the function of mitochondria, creating a vicious cycle that exacerbates the degree of damage to testicular tissue [30]. Membrane potential (ΔΨm) is established on both sides of the mitochondrial inner membrane by electron transfer in the mitochondrial respiratory chain, which performs several important functions, including driving ATP synthesis and aiding in the transport of mitochondrial precursor proteins and some metabolites across the inner membrane [31, 32]. Many of these factors work together on Leydig cells, ultimately leading to decreased androgen production, which may also be a cause of infertility. Furthermore, existing literature has established a link between mitochondrial dysfunction and cellular senescence [33] with evidence suggesting that mitochondrial ROS and the mitochondrial membrane potential play a role in retrograde signalling [34] pathways associated with ageing. This has been observed in various cell types, including those found in breast cancer and hepatocellular carcinoma. In summary, *COX5B* expression was decreased in surgically modelled cryptorchid SD rats and low expression of *COX5B* in the TM3 cell line resulted in mitochondrial dysfunction, such as decreased ATP production, decreased MMP, and increased ROS production leading to increased apoptosis, thus inhibiting cell proliferation. It suggests that cryptorchidism indeed impairs mitochondrial function in Leydig cells by affecting the expression of *COX5B*, leading to diminished fertility.

There are still some limitations in this experiment; for the acquisition method of the cryptorchid rat model, a surgical‐mechanical modelling method was chosen, although there is a certain difference with the clinical neonatal congenital cryptorchidism and the existing studies on cryptorchidism mostly used flutamide for drug induction. Flutamide was not chosen because of its direct effect on mitochondria [35]. In subsequent studies, we can first further explore whether the reversal of *COX5B* low expression in cryptorchid testis can restore the normal function of Leydig cells to verify its feasibility as a therapeutic target. Furthermore, multiple indicators related to mitochondrial dysfunction will be comprehensively analysed to construct a more comprehensive mitochondrial function assessment system, which will provide a more solid foundation for an in‐depth understanding of cryptorchidism pathogenesis.

To conclude, the abnormal expression of *COX5B* in cryptorchidism indicates the possibility of a new therapeutic target, and it may be a new therapeutic idea to reduce the pathological damage of testicular tissues by inhibiting the decrease in the expression of *COX5B*, thus maintaining the normal function of mitochondria.

## Author Contributions

**Xuehan Wang:** writing – original draft (equal). **Dashuai Miao:** writing – original draft (equal). **Songyi Ye:** writing – review and editing (equal). **Hua Xian:** writing – review and editing (equal). **Wenliang Ge:** writing – review and editing (equal).

## Conflicts of Interest

The authors declare no conflicts of interest.

## Data Availability

Data supporting the findings of this study are available on reasonable request from the appropriate authors.
